# Study on the Performance and Mechanism of Cement Solidified Desulfurization Manganese Residue

**DOI:** 10.3390/ma16114184

**Published:** 2023-06-04

**Authors:** Shicheng Wang, Fang Wang, Jialing Che, Lihua Ma

**Affiliations:** 1 School of Civil and Hydraulic Engineering, Ningxia University, Yinchuan 750021, China; wangshichengvictory@stu.nxu.edu.cn (S.W.); che_jialing@nxu.edu.cn (J.C.); 2 Ningxia Tianyuan Manganese Industry Group Co., Ltd., Zhongwei 755100, China; mlharl@163.com

**Keywords:** desulfurization manganese residue, ordinary portland cement, curing, flexural strength, compressive strength, leaching toxicity, mechanism analysis

## Abstract

Desulfurized manganese residue (DMR) is an industrial solid residue produced by high-temperature and high-pressure desulfurization calcination of electrolytic manganese residue (EMR). DMR not only occupies land resources but also easily causes heavy metal pollution in soil, surface water, and groundwater. Therefore, it is necessary to treat the DMR safely and effectively so that it can be used as a resource. In this paper, Ordinary Portland cement (P.O 42.5) was used as a curing agent to treat DMR harmlessly. The effects of cement content and DMR particle size on flexural strength, compressive strength, and leaching toxicity of a cement-DMR solidified body were studied. The phase composition and microscopic morphology of the solidified body were analyzed by XRD, SEM, and EDS, and the mechanism of cement-DMR solidification was discussed. The results show that the flexural strength and compressive strength of a cement-DMR solidified body can be significantly improved by increasing the cement content to 80 mesh particle size. When the cement content is 30%, the DMR particle size has a great influence on the strength of the solidified body. When the DMR particle size is 4 mesh, the DMR particles will form stress concentration points in the solidified body and reduce its strength. In the DMR leaching solution, the leaching concentration of Mn is 2.8 mg/L, and the solidification rate of Mn in the cement-DMR solidified body with 10% cement content can reach 99.8%. The results of XRD, SEM, and EDS showed that quartz (SiO_2_) and gypsum dihydrate (CaSO_4_·2H_2_O) were the main phases in the raw slag. Quartz and gypsum dihydrate could form ettringite (AFt) in the alkaline environment provided by cement. Mn was finally solidified by MnO_2_, and Mn could be solidified in C-S-H gel by isomorphic replacement.

## 1. Introduction

Manganese is an important industrial metal used as an additive in the production of various steels, alloys, electronic components, and special chemicals [1]. More than 98% of the world’s electrolytic manganese metal is produced in China [2]. According to statistics, 8~12 tons of EMR will be produced for every ton of manganese metal produced [3,4,5,6,7,8,9], and the accumulated stock of EMR in China has reached hundreds of millions of tons. At present, most electrolytic manganese enterprises transport EMR to the storage yard for wet storage, which occupies a lot of land resources, and at the same time, there are serious environmental pollution and safety risks [10]. Under the action of leaching and rainwater leaching, manganese and ammonia nitrogen in surface water will exceed the standard, which will cause great harm to the environment. Besides, EMR also contains a small amount of Cr, Cu, Ni, Pb, and As. The research shows that the leaching concentrations of manganese and ammonia nitrogen reach 912.34 mg/L and 1030 mg/L, respectively, far exceeding the values specified in the reference standard [11,12,13]. Moreover, heavy metals in EMR will migrate to rivers, lakes, and groundwater during stacking, which will cause serious environmental pollution and endanger human health [14,15]. Therefore, reasonable and effective treatment of EMR to minimize the harm of manganese slag has become an urgent problem to be solved in the electrolytic manganese industry.

In order to reduce the harm of EMR, many scholars have done a lot of in-depth research. Solidification/stabilization technology is one of the main means of harmlessly treating toxic and harmful solid waste at home and abroad. By adding cementitious materials to wrap toxic pollutants, pollutants are not easy to leach. Liu et al. [16] used slaked lime, carbide slag, and red mud to remove ammonia nitrogen in EMR, and the maximum removal rate of ammonia nitrogen reached 98.2%. Chen et al. [17] solidified EMR by carbonizing active magnesium oxide, which not only improved the strength of EMR but also reduced the content of heavy metals in EMR. Zhan et al. [18] calcined the composite alkali activator and added alkali and EMR together, which improved the curing efficiency of heavy metals in MSWI fly ash and EMR. Zhang et al. [19] prepared road base materials with red mud, carbide slag, and blast furnace slag as stabilizers/curing agents (S/S). The results show that Mn^2+^ can be cured well when the dose of S/S reaches 20%. Wang et al. [20] combined cement solidified waste with direct electric solidification. The results showed that with the increase in voltage, the leaching amount of Mn^2+^ and NH_4_^+^-N in cement-EMR slurry decreased, and its leaching concentration met the Chinese standard (2 mg/L). Zhang et al. [21] used lime and fly ash as stabilizers and cement as a curing agent to stabilize manganese slag and studied the stabilization effects of Mn and Pb in manganese slag. When the dosage of quicklime is 2.5%, the dosage of fly ash is 3%, and the dosage of cement is 12%, the best effect is achieved, and the leaching concentration of Mn and Pb meets the requirements of a Class III water source in China’s surface water environmental quality standard. Chen et al. [22] solidified manganese slag samples with fly ash, quicklime, and cement and compared the solidification effects. The test results showed that the solidification effects of the three solidification materials on soluble Mn were remarkable. Zhang et al. [23] used alkaline substances in red mud to treat ammonia nitrogen and soluble manganese ions in EMR, which could solidify heavy metal ions in waste residue under suitable treatment conditions. Shu et al. [24] used magnesium oxide and different phosphorus sources to stabilize and solidify Mn^2+^ and NH_4_^+^-N in EMR simultaneously (S/S). When the molar ratio of Mg: P was 3:1 and the dosage of PM was 8 wt%, the S/S efficiency of Mn^2+^ and NH_4_^+^-N reached 91.58% and 99.98%, respectively. Lan et al. [25] invented a new method to effectively recover ammonium nitrogen and solidify Mn and other heavy metals by mechanical grinding in the presence of water and calcium oxide. Its research shows that the solidification/recovery efficiency of Mn and ammonia nitrogen can reach more than 98%. Shu et al. [26] used a new basic combustion raw material (BRM) to stabilize/solidify Mn^2+^ and NH_4_^+^-N in EMR. The leaching test results showed that the concentration of heavy metals was within the allowable range of the standard. Mn and NH_3_-N are considered the main harmful elements in EMR [27,28,29]. High temperatures and high pressures can convert NH_3_-N in EMR into NH_3_ and N_2_. NH_3_ is recovered to make ammonia water, and N_2_ is discharged into the air. In addition, calcination can effectively reduce the leaching hazards of heavy metals such as Mn, Pb, Cr, and As in EMR. In order to dispose of EMR and reduce its harm to the environment, EMR was calcined at high temperature and pressure and desulfurized to prepare DMR. Compared with EMR, DMR does not contain ammonia nitrogen, and heavy metals are not easy to leach. However, it is unknown whether there is a risk of heavy metal leaching from the large stockpile of DMR.

Based on this, this paper uses cement curing agents to treat DMR, so that the harmful heavy metals in manganese slag are solidified. P.O 42.5 cement was selected to solidify DMR with different particle sizes and dosages. By testing the flexural strength, compressive strength, and leaching toxicity of the solidified body, the effects of cement dosage and DMR particle size on mechanical strength and leaching toxicity were analyzed. The comprehensive micromorphology and elements of the solidified body were analyzed by XRD, SEM, and EDS, and the mechanism of cement solidification in DMR was discussed. The research in this paper can provide a reference for the harmless treatment and resource utilization of DMR.

## 2. Materials and Methods

### 2.1. Materials and Instruments

Desulfurized manganese residue (DMR) is taken from a stockyard of Ningxia Tianyuan Manganese Industry Group Co., Ltd., Ningxia, China; cement is taken from Horse Racing Cement Plant (P.O 42.5); sulfuric acid (H_2_SO_4_) and nitric acid (HNO_3_) were purchased from Ningxia Dazheng Chemical Co., Ltd. (Ningxia, China), both of which were analytically pure. The water is distilled water.

**Figure 1:**
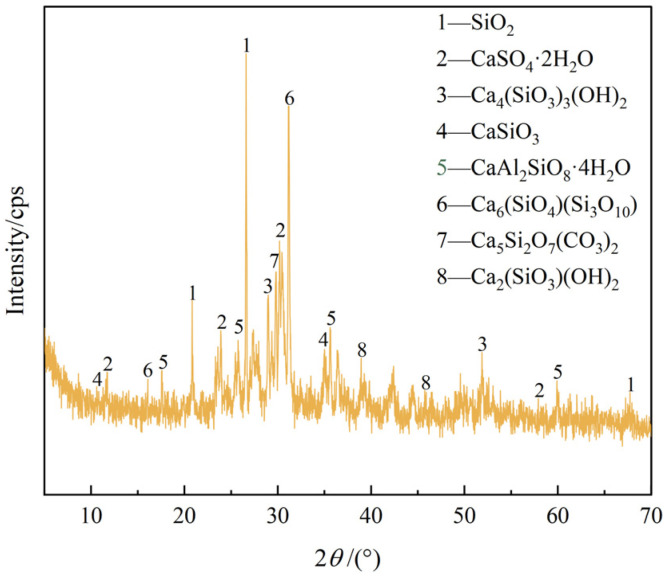
XRD pattern of DMR.

**Figure 2:**
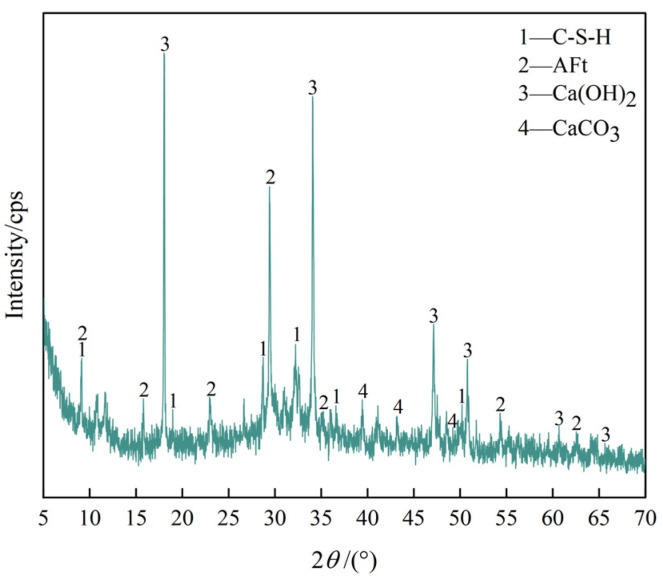
XRD pattern of cement net paste 28d solidified body.

An X-ray fluorescence spectrometer (XRF, Netherlands PANalytical Axios Co., Ltd., Almolo, The Netherlands) was used to analyze the main chemical components of DMR and cement, and the results are shown in Table 1. An X-ray diffractometer (XRD) was used to analyze DMR and the main minerals of cement. The results are shown in Figure 1 and Figure 2.

As can be seen from Table 1 and Figure 1, DMR is not a simple mineral but consists of quartz, gypsum, orthorhombic wollastonite, and other minerals. The content of gypsum (SO_4_^2−^) is high, and ettringite (Aft) can be formed in an alkaline environment [30]. The hydration reaction of tricalcium silicate and dicalcium silicate in cement components is as shown in Formulas (1) and (2), which can produce xCaO·SiO_2_·yH_2_O(C-S-H), and the Ca(OH)_2_ can provide a good alkaline environment for cement-DMR solidified bodies. In addition, the diffraction peaks of SiO_2_, Ca_6_(SiO_4_)(Si_3_O_10_), and CaSO_4_·2H_2_O are sharp, which indicates that they have high crystallinity and large crystalline grains, and the content of silicate compounds in DMR is high, so they belong to industrial waste residues with high silicate content.

(1) $$3\mathrm{CaO}\cdot {\mathrm{SiO}}_{2}+nH_{2}O=x\mathrm{CaO}\cdot {\mathrm{SiO}}_{2}\cdot yH_{2}O+(3-x{)\mathrm{Ca}(\mathrm{OH})}_{2}$$

(2) $$2\mathrm{CaO}\cdot {\mathrm{SiO}}_{2}+nH_{2}O=x\mathrm{CaO}\cdot {\mathrm{SiO}}_{2}\cdot yH_{2}O+(2-x{)\mathrm{Ca}(\mathrm{OH})}_{2}$$

As can be seen from Figure 2, the hydration products of cement for 28d are mainly ettringite, calcium hydroxide, hydrated calcium silicate, and a small amount of calcium carbonate. At 39.37°, the diffraction peak of calcium carbonate is sharp, and then the intensity gradually decreases, which is due to the absorption of carbon dioxide in the air by calcium hydroxide to generate calcium carbonate. With the reaction, the amount of calcium hydroxide gradually decreases, and the amount of calcium carbonate also decreases gradually.

### 2.2. Pretreatment of DMR

**Figure 3:**
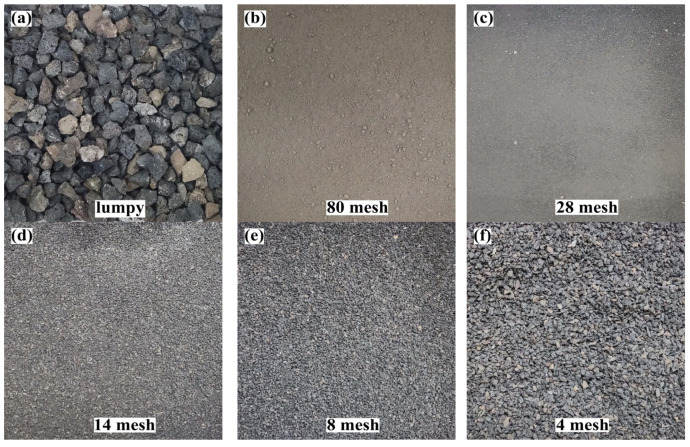
DMR preprocessing renderings: (**a**) Lumpy DMR; (**b**) 80 mesh DMR; (**c**) 28 mesh DMR; (**d**) 14 mesh DMR; (**e**) 8 mesh DMR; (**f**) 4 mesh DMR.

The bulk DMR (Figure 3a) was dried at 105 °C for 48 h. After cooling, crushing, grinding, and sieving, five DMR with different particle sizes—80 mesh, 28 mesh, 14 mesh, 8 mesh, and 4 mesh—were obtained, respectively. The treatment effect is shown in Figure 3b–f.

### 2.3. Preparation of Cement Solidified DMR Solidified Body

The larger the specific surface area is, the more active sites can be provided for the hydration reaction, and the reaction is more sufficient. Therefore, 80 mesh DMR with a larger specific surface area is selected to study the influence of different cement content on the cement-DMR solidified body; Fang et al. [31] obtained 80 mesh EMR under 30% cement content, and the strength of the solidified body exceeded 10 MPa to meet the requirements of comprehensive utilization. Therefore, the influence of DMR with different particle sizes on the cement-DMR solidified body was studied under 30% cement content. The specific test scheme is as follows:

(1) The influence of different cement content: the ratio of cement to 80 mesh DMR is 10%, 15%, 20%, 25%, 30%, 35%, 40%, 45%, 50%, and the water cement ratio is 0.44;
(2) Put the cement and DMR into a mixer and mix them at low speed for 1 min, then add water to the mixture slowly and stir for 3 min, and then inject them into a test mold of 40 mm × 40 mm × 160 mm after mixing evenly. Vibrate the cement mortar on the shaking table for 120 times until it is compact, and smooth it with a scraper after vibrating. After curing at 22 °C and 96% humidity for 24 h, demoulding was carried out, and curing was continued to the specified curing ages of 3d, 7d, 14d, and 28d under the above conditions, and the cement-DMR solidified specimens to be tested were obtained.

### 2.4. Flexural Strength and Compressive Strength

According to GB/T 17671-2021 [32], the flexural strength and compressive strength of cement-DMR solidified body specimens for 3d, 7d, 14d and 28d were tested by an electronic pressure testing machine controlled by a microcomputer (Hangzhou Xin Hi-Tech Co., Ltd., Hangzhou, China). The loading rate of flexural strength is 50 N/s, the loading rate of compressive strength is 2400 N/s, and the average value is taken for each group of three samples.

### 2.5. Characterization of Solidified Body

The main components of the cured body after the hydration reaction were analyzed by an X-ray diffractometer (Germany BRUKER AXS Co., Ltd., Karlsruhe, Germany), and the cured body was milled through a 200 mesh sieve hole for scanning, the scanning range was 5~70°, and the scanning rate was 2 (°)/min; Scanning electron microscope-energy dispersive spectroscopy (SEM-EDS, Germany Carl Zeiss Co., Ltd., Oberkochen, Germany) was used to analyze the microscopic morphology and main elements of the cured body.

### 2.6. Leaching Toxicity Test

Leaching toxicity test according to HJ 557-2010 [33]. The leached toxic specimens were cured to the specified age, and after being crushed, they were sampled and dried at 105 °C and passed through a 3 mm sieve. A total of 10 g of sample was weighed and placed in a 250 mL flask, and 100 g of extractant (mixed sulfuric acid and nitric acid) was added. The cap of the flask was tightly sealed and placed in a speed-regulating, multi-purpose oscillator. The flask was shaken at (25 ± 2) °C at a frequency of (110 ± 10 oscillations/min) for 8 h, and then the flask was removed. After standing for 16 h, the concentration of heavy metal elements was measured by triple quadrupole ICP-MS (Japan Agilent Technologies Co., Ltd., Tokyo, Japan), and the ammonia nitrogen content was measured by a UV-VIS spectrophotometer (Shanghai Aoyi Instrument Co., Ltd., Shanghai, China).

## 3. Results and Discussion

### 3.1. Flexural Strength and Compressive Strength

**Figure 4:**
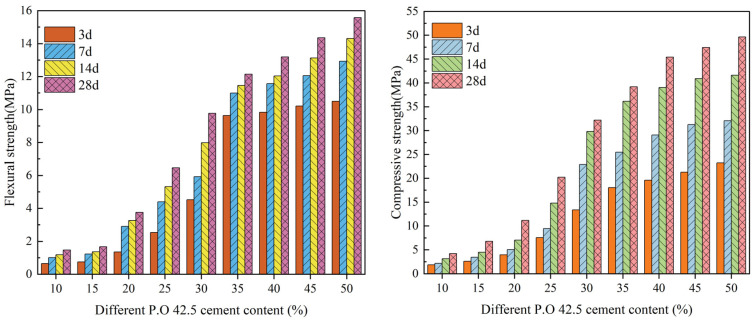
Flexural strength and compressive strength of the solidified DMR body at different ages with different P.O 42.5 content.

Figure 4 shows the test results for flexural strength and compressive strength of a cement-DMR solidified body under different cement content and different age conditions with an 80 mesh DMR ratio.

As can be seen from Figure 4, with the increase in cement content and curing time, the flexural and compressive strengths of the solidified body increase. When the cement content is 10~15%, the flexural strength and compressive strength increase slowly, but when the cement content reaches 15%, the compressive strength increases rapidly. The flexural strength and compressive strength are the highest at each age when the cement content is 50%. The flexural strengths of 3d, 7d, 14d, and 28d are 10.5 MPa, 12.9 MPa, 14.3 MPa, and 15.6 MPa, respectively. which is 17.5, 12.9, 11.9, and 10.4 times that of 3d, 7d, 14d, and 28d, respectively, when the cement content is 10%. The compressive strength is 23.2 MPa, 32.1 MPa, 41.6 MPa, and 49.6 MPa, respectively, which is 12.2, 14.6, 13.4, and 11.8 times of the 3d, 7d, 14d, and 28d strengths when the cement content is 10%. The results show that more hydrated calcium silicate gel (C-S-H) can be formed in the cement-DMR solidified body with higher cement content [34], and more ettringite (AFt) can be formed in the alkaline environment of cement hydration due to a large amount of gypsum dihydrate (CaSO_4_·2H_2_O) in the DMR solidified body. In addition, DMR is obtained by desulfurization and deamination calcination of EMR at high temperature and pressure. It is mainly a solid calcined slag with low-temperature inert phases such as cristobalite, calcium sulfate, and silicates such as calcium and aluminum as main phases and has high activity [35]. In addition, DMR itself has weak volcanism and promotes the hydration reaction of cement [36]. To sum up, it is precisely because of the enhancement of the hydration reaction, the increase of hydration products, and the formation of more C-S-H and AFt that the flexural strength and compressive strength of the solidified body at each age are significantly improved under 50% cement content.

**Figure 5:**
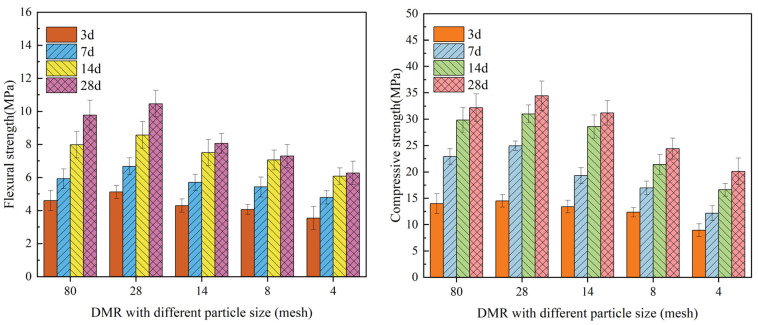
Flexural strength and compressive strength of the DMR solidified body with different particle sizes at different ages under 30% P.O 42.5 content.

Figure 5 shows the test results for flexural strength and compressive strength of a cement-DMR solidified body with different particle sizes at 30% cement content.

As can be seen from Figure 5, the particle size of DMR has a great influence on the strength of the cured body. The flexural strength and compressive strength of the cured body with DMR particle size of 28 mesh are the highest, and the flexural strength of the cured body with DMR particle size of 80 mesh is increased by 10.9%, 13.6%, 7.5%, and 7.1% for 3d, 7d, 14d, and 28d, respectively. The compressive strength increased by 3.6%, 8.7%, 4.0%, and 6.8%, respectively. The flexural strength and compressive strength of the 80 mesh DMR solidified body at 28d were 9.8 MPa and 32.2 MPa, respectively, and the flexural strength and compressive strength of the 28 mesh DMR solidified body at 28 mesh size were 10.5 MPa and 34.4 MPa, respectively. It can be seen that with the slight increase of particle size, DMR with slightly larger particles acted as a part of the aggregate in the hardened body, so the strength increased slightly. However, with the increase in particle size, the flexural strength and compressive strength of the DMR solidified body decreased. The flexural strength and compressive strength of the DMR solidified body at 28d with 30% cement content and 4 mesh particle size were 6.3 MPa and 20.1 MPa, respectively, which decreased by 40.0% and 41.6%, respectively, compared with 28 mesh. The analysis shows that when the particle size is large, the DMR in the middle of the agglomerated particles cannot be fully mixed with cement uniformly, forming a failure stress concentration point similar to honeycomb, and the uneven stress distribution during flexural and compressive resistance leads to stress concentration, which reduces the strength of the solidified body. In addition, DMR has certain pozzolanic properties, and the finer the particle size, the more obvious the effect, which can promote the strength of a cement-DMR-solidified body. At the same time, when DMR is ground to a certain extent, its surface area is larger. When the fine DMR powder acts as a physical filling, it can be more effectively dispersed into the pores of the cured body, which improves the pore structure of the cured body, makes its structure denser, and shows greater macroscopic strength.

### 3.2. Toxicity Leaching Test of Cement Solidified Desulfurization Manganese Residue

The content of heavy metals in DMR was determined by triple quadrupole ICP-MS, and the content of ammonia nitrogen was measured by a UV-VIS spectrophotometer. The test results are shown in Table 2. The concentrations of Mn and NH_4_-N in the leaching solution of DMR are 2.8 mg/L and 0 mg/L, respectively, which are 1.4 times and 0 times the Class I emission standard in GB8978-1996 [37]. Only the leaching concentration of Mn slightly exceeds the standard, and other heavy metals such as Pb, Ni, Cu, and Zn are far lower than the requirements of GB8978-1996. Therefore, Mn is the pollutant that needs attention in DMR, and the concentration of Mn in the solidified body is mainly determined. The reason why ammonia nitrogen is not detected is that ammonia nitrogen is decomposed into ammonia and nitrogen at high temperatures and pressures. Ammonia is collected for manufacturing ammonia water, while nitrogen is discharged into the air. It can be seen that ammonia nitrogen in EMR can be effectively removed by high-temperature and high-pressure calcination.

**Figure 6:**
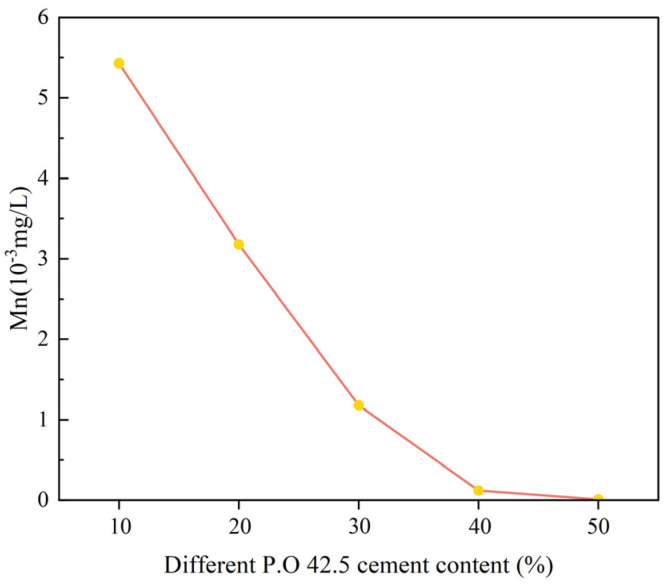
Leaching toxicity of 28d solidified body with different P.O 42.5 content.

**Figure 7:**
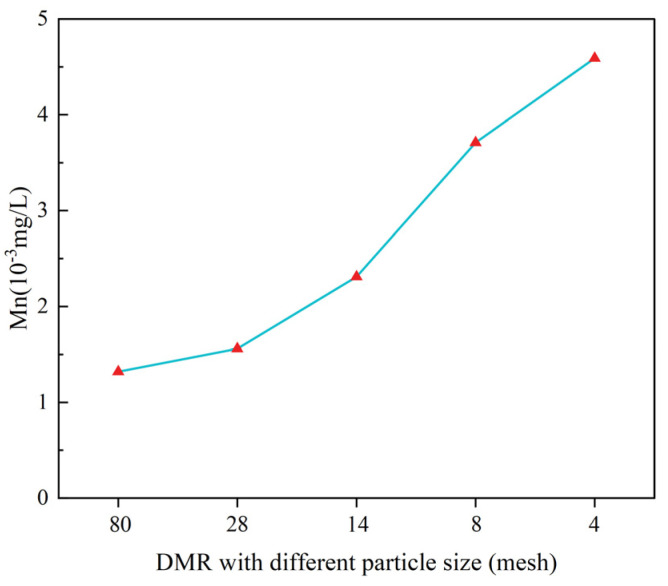
Leaching toxicity of 28d solidified body of DMR with different particle sizes at 30% P.O 42.5 content.

Figure 6 and Figure 7 are Mn leaching concentration of cement-DMR solidified body at 28d.

As can be seen from Figure 6 and Figure 7, the content of leaching toxicity decreases with the increase in cement content and increases with the increase in particle size. The concentration of Mn is 5.43 × 10^−3^ mg/L and 0.01 × 10^−3^ mg/L when the cement content is 10% and 50%, and the solidification rate of Mn by cement is over 99.8%. The concentrations of Mn in 80 mesh and 4 mesh solidified bodies for 28d were 1.32 × 10^−3^ mg/L and 4.59 × 10^−3^ mg/L, respectively. With the increase in DMR particle size, the curing effect of Mn decreases slightly, but all of them can meet the Class I emission standard in GB8978-1996. There are three main reasons for the low leaching concentration of Mn in cement-DMR-solidified bodies. One is the high alkalinity and strong acid retarding ability of DMR itself; second, the cement hydration reaction provides a better alkaline environment and physical encapsulation; third, the hydration products produced by cement solidification of DMR have a strong acid retarding ability, and a large amount of acid is needed to leach Mn from DMR [36].

To sum up, cement can effectively solidify soluble Mn in DMR, and increasing the content of cement can further remove Mn; DMR particles with larger particle sizes are not as good as DMR particles with smaller particle sizes. The reason for the analysis is that the specific surface area of DMR particles with a larger particle size is smaller. In contrast, DMR particles with a smaller particle size and larger specific surface area can participate in hydration reaction more fully, have a better curing effect, and lower leaching toxicity. Combined with XRD spectrum analysis of DMR, it can be seen (Figure 1) that the main components of DMR are low temperature cristobalite and gypsum, and the heavy metal Mn may have been wrapped by the combined crystals of quartz, calcium sulfate and other minerals melted at high temperature, and melted with other metal elements to transform into silicate crystals, finally forming stable silicate compounds. The leaching toxicity of DMR obtained by high temperature and high-pressure desulfurization calcination treatment has been significantly reduced, and the Mn in DMR can be further stabilized by cement solidification.

### 3.3. Study on Hydration Products and Micro-Morphology Analysis of Cement Solidified Desulfurization Manganese Residue Solidified Body

#### 3.3.1. XRD Analysis

**Figure 8:**
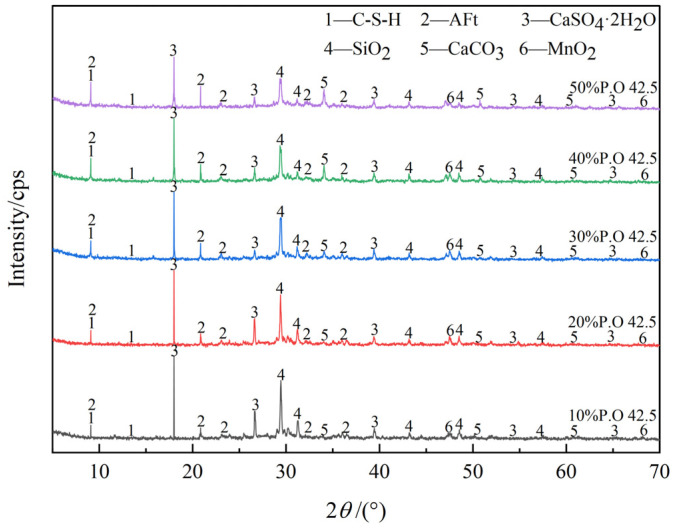
XRD patterns of 28d solidified body with different P.O 42.5 content.

Figure 8 is the XRD pattern of DMR with 80 mesh particle size solidified body at 28d under different cement content.

As can be seen from Figure 8, with the increase in cement content, the intensity of the diffraction peaks of the gypsum dihydrate phase (CaSO_4_·2H_2_O) and quartz phase (SiO_2_) decreases obviously. Analysis shows that in an alkaline environment, quartz and gypsum dihydrate can be activated to promote the formation of hydration products, and OH^-^ leads to the fracture of chemical bonds between Al-O and Si-O. At the same time, DMR provides a high sulfate environment, combining Ca^2+^ and SO_4_^2−^ to form Aft [38]. The main reactions are shown in Formulas (3)–(6), and the reactions shown in Formulas (3)–(6) consume OH^−^, which promotes the forward hydration reaction of Portland cement as shown in Formulas (1) and (2). With the increase in cement content, the alkaline environment of the whole system is stronger, and the content of hydration products C-S-H and Aft increases, which has a positive effect on strength formation.

It can be seen from Figure 8 that the diffraction peaks of AFt and C-S-H are at 9.1°. with the increase of cement content, and the peak is constantly sharp, which explains that it has higher early strength under high cement content. A small amount of soluble Mn in DMR combined with the OH^−^ ion in an alkaline environment, and soluble Mn finally formed stable and insoluble MnO_2_ [19].

(3) $${\mathrm{SiO}}_{2}{+\mathrm{OH}}^{-}{+H}_{2}O\to {\left[H_{3}{\mathrm{SiO}}_{4}\right]}^{-}$$

(4) $${\mathrm{Al}}_{2}O_{3}{+2\mathrm{OH}}^{-}\to 2{\mathrm{AlO}}_{2}^{-}+H_{2}O$$

(5) $${\mathrm{AlO}}_{2}^{-}+{\mathrm{OH}}^{-}+H_{2}O\to {\left[H_{3}{\mathrm{AlO}}_{{}^{4}}\right]}_{2}^{-}+{\left[{\mathrm{Al}(\mathrm{OH})}_{6}\right]}_{3}^{-}$$

(6) $${\left[\mathrm{Al}{\left(\mathrm{OH}\right)}_{6}\right]}_{3}^{-}+{\mathrm{Ca}}^{+}+{\mathrm{SO}}_{4}^{2-}+H_{2}O\to {\mathrm{Ca}}_{6}{\mathrm{Al}}_{2}{\left({\mathrm{SO}}_{4}\right)}_{3}{\left(\mathrm{OH}\right)}_{12}\cdot xH_{2}O(\mathrm{Aft})$$

#### 3.3.2. SEM-EDS Analysis

**Figure 9:**
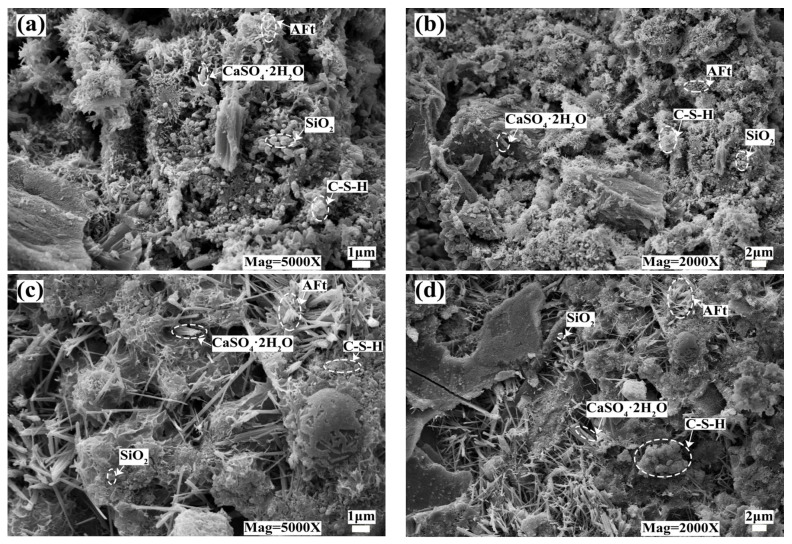
SEM micrographs of 28d solidified body with different P.O 42.5 content: (**a**) 10% P.O 42.5; (**b**) 10% P.O 42.5; (**c**) 50% P.O 42.5; (**d**) 50% P.O 42.5.

Figure 9 shows the SEM micrographs of the 28d solidified body with an 80 mesh particle size DMR ratio of 10% and 50% cement content.

As can be seen from Figure 9, ettringite (Aft) crystal is needle-rod shape, C-S-H gel is fibrous shape [39,40], silicon dioxide crystal (SiO_2_) is a spherical particle, and gypsum crystal (CaSO_4_·2H_2_O) is rod shape in the cured body, among which AFt crystal is the main source of strength in the cured body. On the other hand, from Figure 9a,b, it can be seen that under low cement content, the solidified body is mainly rod-like gypsum crystal and spherical silica crystal, with less C-S-H gel, and the solidified body has obvious pores, which explains why the compressive strength of the solidified body at 10% cement content for 28d is low. With the increase in cement content, the C-S-H gel and Ca(OH)_2_ content of the hydration reaction increased, which promoted the formation of AFt. As can be seen from Figure 9c,d, the flocculent C-S-H gels are closely aggregated and connected to each other [41], showing needle-bar ettringite crystals, which are closely overlapped to form a dense network structure and jointly improve the mechanical strength [18], which is consistent with the results of flexural strength and compressive strength shown in Section 2.1.

**Figure 10:**
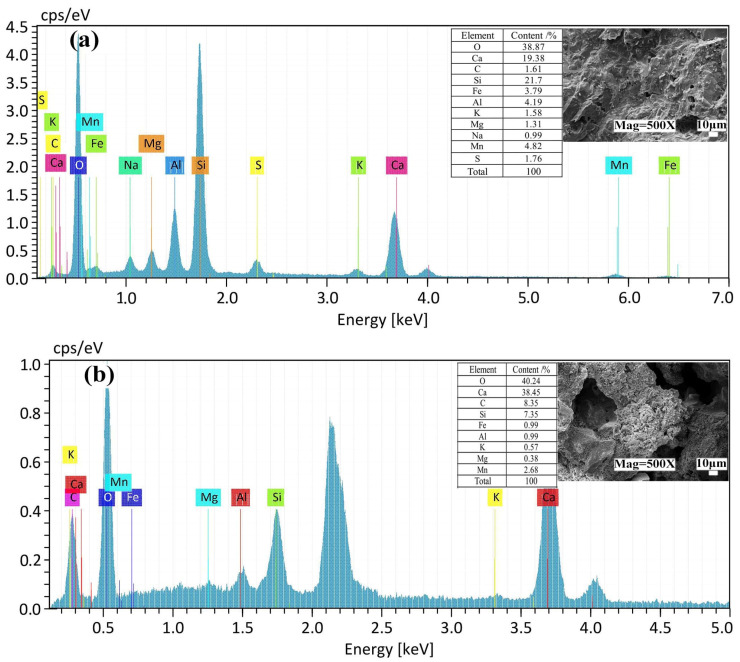

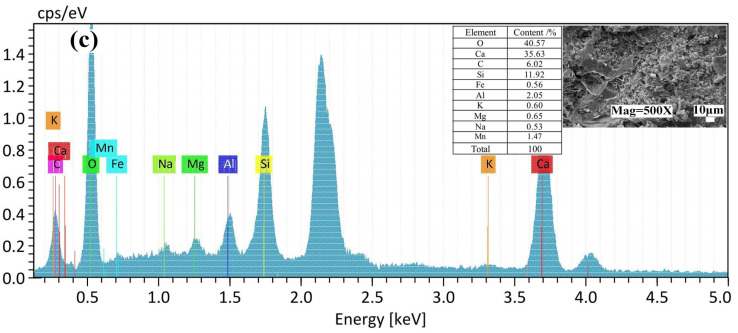
SEM-EDS spectrum of 28d solidified body with DMR and different P.O 42.5 content: (**a**) SEM-EDS diagram of DMR; (**b**) SEM-EDS diagram of the solidified body with 10% P.O 42.5 content; (**c**) SEM-EDS diagram of the solidified body with 50% P.O 42.5 content.

Figure 10 is the element analysis of the SEM-EDS spectrum of 28d solidified body under raw slag, 80 mesh particle size DMR mixed with 10% and 50% cement.

It can be seen from Figure 10 that the main elements in DMR raw slag are Ca, Si, Fe, Al, K, Mg, and Mn. Combined with SEM, it can be seen that the crystal phase in the raw slag is distinct, and it shows that the main phases at the sample point are SiO_2_ and CaSO_4_·2H_2_O. At the same time, the distribution of Ca and S proves the existence of CaSO_4_·2H_2_O. Combined with the SEM images of Figure 10b,c and the distribution of elements, C-S-H gel is formed with Ca, Si, and O as the main elements, and there is metal Mn in the cured C-S-H gel, which is due to the isomorphic transformation during the formation of layered C-S-H gel, and Mn can undergo displacement reactions with Ca, Al, Si, and Fe plasma in its lattice [42], thus immobilizing the lattice into the crystal structure and existing in a more stable form. This also explains that the leaching toxicity of Mn is significantly reduced after DMR is cured by cement.

## 4. Conclusions

In this paper, P.O 42.5 cement was used to solidify DMR. Through the tests of flexural strength, compressive strength, and leaching toxicity, the effects of cement content and DMR particle size on the mechanical strength and leaching toxicity of the cement-DMR-solidified body were analyzed. At the same time, the DMR mechanism of cement solidification was analyzed by means of XRD, SEM, and EDS. The following conclusions are drawn:

(1) DMR, prepared by high-temperature and high-pressure desulfurization and deamination calcination, has a solidified body mixed with cement that has good flexural and compressive strength. Under 80 mesh particle size, the increase in cement content can significantly improve the flexural strength and compressive strength of the solidified body at different ages. Under the condition of 30% cement content, DMR with different particle sizes has a great influence on the strength of the solidified body, and the solidified body formed by 30% cement with four mesh particle sizes will form stress concentration points, thus reducing the strength of the solidified body.
(2) Cement can effectively solidify the soluble Mn in DMR. With the increase in cement content, the Mn in DMR can be further removed. The solidification rate of Mn in a cement-DMR-solidified body with 10% cement content is 99.8%. The increase in particle size has little negative impact on the solidification of Mn, but it can still meet the Class I emission standard in GB8978-1996.
(3) C-S-H gel is formed by the cement hydration reaction, and DMR and cement can provide enough Al elements. Under the alkaline environment provided by the cement hydration reaction, the main substances of DMR, dihydrate gypsum (CaSO_4_·2H_2_O) and quartz (SiO_2_), react to form ettringite (AFt), and C-S-H gel and AFt cooperate to form a dense network structure, which improves the strength of the solidified body. In the alkaline environment formed by cement hydration, a small amount of soluble Mn in DMR finally forms MnO_2_, and some manganese is solidified in C-S-H gel through isomorphic transformation.

## Figures and Tables

**Table 1:** DMR and Main Chemical Compositions of Cement (%).

Composition	SiO_2_	CaO	Al_2_O_3_	MnO	Fe_2_O_3_	MgO	SO_3_	Na_2_O	K_2_O	Others
DMR	40.22	31.14	7.98	6.63	6.41	1.89	1.36	1.31	1.25	1.81
P.O 42.5	22.35	59.22	6.49	-	2.73	4.01	2.22	-	-	1.78

**Table 2:** Test results of leaching toxicity of DMR.

Test Elements	Leaching Mass Concentration/(mg·L^−1^)	GB8978-1996/(mg·L^−1^)
Mn	2.8	2.0
Pb	0.003	1.0
Ni	0.015	1.0
Cu	0.004	1.0
Zn	0.013	5.0
Se	0.009	0.2
As	0.175	0.5
P	0.026	0.3
Cd	0.0001	0.1
Cr	0.0009	1.5
NH_4_-N	N.D ^1^	15

^1^ is not detected.

## Data Availability

The data presented in this study are available on request from the corresponding author.

## References

[1] Lu J.M., Dreisinger D., Glück T. (2014). Manganese electrodeposition—A literature review. Hydrometallurgy.

[2] Zhan X.Y., Wang L.A., Wang L., Gong J., Wang X., Song X., Xu T.T. (2021). Co-sintering MSWI fly ash with electrolytic manganese residue and coal fly ash for lightweight ceramisite. Chemosphere.

[3] Shu J.C., Chen M.J., Wu H.P., Li B.B., Wang B., Li B., Liu R.L., Liu Z.H. (2019). An innovative method for synergistic stabilization/solidification of Mn^2+^, NH_4_^+^-N, PO_4_^3−^ and F^−^ in electrolytic manganese residue and phosphogypsum. J. Hazard. Mater..

[4] Shu J.C., Liu R.L., Liu Z.H., Chen H.L., Tao C.Y. (2016). Simultaneous removal of ammonia and manganese from electrolytic metal manganese residue leachate using phosphate salt. J. Clean. Prod..

[5] He D.J., Shu J.C., Zeng X.F., Wei Y.F., Chen M.J., Tan D.Y., Liang Q. (2022). Synergistic solidification/stabilization of electrolytic manganese residue and carbide slag. Sci. Total. Environ..

[6] Li J., Sun P., Li J.X., Lv Y., Ye H.P., Li S., Du D.Y. (2020). Synthesis of electrolytic manganese residue-fly ash based geopolymers with high compressive strength. Constr. Build. Mater..

[7] He D.J., Shu J.C., Wang R., Chen M.J., Wang R., Gao Y.S., Liu R.L., Liu Z.H., Xu Z.H., Tan D.Y. (2021). A critical review on approaches for electrolytic manganese residue treatment and disposal technology: Reduction, pretreatment, and reuse. J. Hazard. Mater..

[8] Zhou Y.X. (2021). Reusing electrolytic manganese residue as an activator: The effect of calcination on its mineralogy and activity. Constr. Build. Mater..

[9] Wang Y.G., Gao S., Liu X.M., Tang B.W., Mukiza E., Zhang N.A. (2019). Preparation of non-sintered permeable bricks using electrolytic manganese residue: Environmental and NH_3_-N recovery benefits. J. Hazard. Mater..

[10] Lan J.R., Sun Y., Huang P., Du Y.G., Zhan W., Zhang T.C., Du D.Y. (2020). Using Electrolytic Manganese Residue to prepare novel nanocomposite catalysts for efficient degradation of Azo Dyes in Fenton-like processes. Chemosphere.

[11] Zheng F., Zhu H., Luo T., Wang H.J., Hou H.B. (2020). Pure water leaching soluble manganese from electrolytic manganese residue: Leaching kinetics model analysis and characterization. J. Environ. Chem. Eng..

[12] Shu J.C., Cai L.H., Zhao J.J., Feng H., Chen M., Zhang X.R., Wu H.P., Yang Y., Liu R.L. (2020). A low cost of phosphate-based binder for Mn^2+^ and NH_4_^+^-N simultaneous stabilization in electrolytic manganese residue. Ecotox. Environ. Saf..

[13] Pang L., Wang D.Q., Wang H., An M.Z., Wang Q. (2022). Occurrence and leaching behaviors of heavy-metal elements in metallurgical slags. Constr. Build. Mater..

[14] Li C.X., Zhong H., Wang S., Xue J.R., Zhang Z.Y. (2015). A novel conversion process for waste residue: Synthesis of zeolite from electrolytic manganese residue and its application to the removal of heavy metals. Colloid. Surface. A.

[15] Li J., Du D.Y., Peng Q.J., Wu C.J., Lv K.L., Ye H.P., Chen S.H., Zhan W. (2018). Activation of silicon in the electrolytic manganese residue by mechanical grinding-roasting. J. Clean. Prod..

[16] Liu X.Y., Ren Y.Y., Zhang Z.Q., Liu X.M., Wang Y.G. (2023). Harmless treatment of electrolytic manganese residue: Ammonia nitrogen recovery, preparation of struvite and nonsintered bricks. Chem. Eng. J..

[17] Chen Z., Fang X.W., Long K.Q., Shen C.N., Yang Y., Liu J.L. (2021). Using the Biocarbonization of Reactive Magnesia to Cure Electrolytic Manganese Residue. Geomicrobiol. J..

[18] Zhan X.Y., Wang L.A., Wang L., Wang X., Gong J., Yang L., Bai J.S. (2019). Enhanced geopolymeric co-disposal efficiency of heavy metals from MSWI fly ash and electrolytic manganese residue using complex alkaline and calcining pre-treatment. Waste Manag..

[19] Zhang Y.L., Liu X.M., Xu Y.T., Tang B.W., Wang Y.G. (2020). Preparation of road base material by utilizing electrolytic manganese residue based on Si-Al structure: Mechanical properties and Mn^2+^ stabilization/solidification characterization. J. Hazard. Mater..

[20] Wang F., Long G.C., Bai M., Wang J.L., Yang Z.H., Zhou X., Zhou J.L. (2022). Cleaner and safer disposal of electrolytic manganese residues in cement-based materials using direct electric curing. J. Clean. Prod..

[21] Zhang S.H., Li X.D., Zhang W., Fan F., Wang Y.Z. (2021). Study on the leaching toxicity and performance of manganese slag-based cementitious materials. Mater. Res. Express..

[22] Chen M., Wei J.M., Jia L.P., Yao Q.Q., Chen Y.S. (2021). Study on Solidification Treatment of Electrolytic Manganese Slag and Numerical Simulation of Slope Stability. Geotech. Geol. Eng..

[23] Zhang J., Li R., Zhang Y., He W.L., Yang J.J., Wang Y. (2023). Study on mutual harmless treatment of electrolytic manganese residue and red mud. Environ. Sci. Pollut. Res..

[24] Shu J.C., Wu H.P., Liu R.L., Liu Z.H., Li B., Chen M.J., Tao C.Y. (2018). Simultaneous stabilization/solidification of Mn^2+^ and NH_4_^+^-N from electrolytic manganese residue using MgO and different phosphate resource. Ecotox. Environ. Saf..

[25] Lan J.R., Zhang S.S., Mei T., Dong Y.Q., Hou H.B. (2021). Mechanochemical modification of electrolytic manganese residue: Ammonium nitrogen recycling, heavy metal solidification, and baking-free brick preparation. J. Clean. Prod..

[26] Shu J.C., Li B., Chen M.J., Sun D.Y., Wei L., Wang Y., Wang J.Y. (2020). An innovative method for manganese (Mn^2+^) and ammonia nitrogen (NH_4_^+^-N) stabilization/solidification in electrolytic manganese residue by basic burning raw material. Chemosphere.

[27] Shu J.C., Liu R.L., Liu Z.H., Chen H.L., Du J., Tao C.Y. (2016). Solidification/stabilization of electrolytic manganese residue using phosphate resource and low-grade MgO/CaO. J. Hazard. Mater..

[28] Wang D.Q., Wang Q., Xue J.F. (2020). Reuse of hazardous electrolytic manganese residue: Detailed leaching characterization and novel application as a cementitious material. Resour. Conserv. Recycl..

[29] Wang D.Q., Wang Q. (2022). Clarifying and quantifying the immobilization capacity of cement pastes on heavy metals. Cement. Concrete. Rec..

[30] Zhang Y.L., Liu X.M., Xu Y.T., Tang B.W., Wang Y.G., Mukiza E. (2019). Synergic effects of electrolytic manganese residue-red mud-carbide slag on the road base strength and durability properties. Constr. Build. Mater..

[31] Fang X.J., Wang Z., Qian J.S., Jiang X.H., Hou P.K. (2010). On the solidification of electrolytic manganese residue with cement and its leaching toxicity. J. Saf. Environ..

[32] (2021). Test Method of Cement Mortar Strength (ISO Method). GB/T 17671-2021.

[33] (2010). Solid Waste—Extraction Procedure for Leaching Toxicity—Horizontal Vibration Method. HJ 557-2010.

[34] Zhang L.F., Yang L., Hao Z.H. (2021). Study on the effect of manganese slag admixture on the properties of cement mortar. Appl. Chem. Ind..

[35] Wang F., Long G.C., Bai M., Wang J.L., Zhou J.L., Zhou X. (2022). Application of electrolytic manganese residues in cement products through pozzolanic activity motivation and calcination. J. Clean. Prod..

[36] Wang D.Q., Fang J.R., Wang Q., Liu Y.J. (2022). Utilizing desulphurized electrolytic-manganese residue as a mineral admixture: A feasibility study. Cement. Concrete. Comp..

[37] (1996). Integrated Wastewater Discharge Standard. GB 8978-1996.

[38] Singh M., Garg M. (1995). Activation of gypsum anhydrite-slag mixtures. Cement. Concrete. Res..

[39] Ren J., Hu L., Dong Z.J., Tang L.P., Xing F., Liu J. (2021). Effect of silica fume on the mechanical property and hydration characteristic of alkali-activated municipal solid waste incinerator (MSWI) fly ash. J. Clean. Prod..

[40] Liu X.M., Zhang N., Yao Y., Sun H.H., Feng H. (2013). Micro-structural characterization of the hydration products of bauxite-calcination-method red mud-coal gangue based cementitious materials. J. Hazard. Mater..

[41] Liu S.H., Ouyang J.Y., Ren J. (2020). Mechanism of calcination modification of phosphogypsum and its effect on the hydration properties of phosphogypsum-based supersulfated cement. Constr. Build. Mater..

[42] Montañés M.T., Sánchez-Tovar R., Roux M.S. (2014). The effectiveness of the stabilization/solidification process on the leachability and toxicity of the tannery sludge chromium. J. Environ. Manag..

